# The Influence of Undercooling ΔT on the Structure and Tensile Strength of Grey Cast Iron

**DOI:** 10.3390/ma14216682

**Published:** 2021-11-05

**Authors:** Józef Dorula, Dariusz Kopyciński, Edward Guzik, Andrzej Szczęsny, Daniel Gurgul

**Affiliations:** 1 Faculty of Foundry Engineering, AGH University of Science and Technology, al. A. Mickiewicza 30, 30-059 Kraków, Poland; Jozef.Dorula@vesuvius.com (J.D.); guz@agh.edu.pl (E.G.); ascn@agh.edu.pl (A.S.); dg@agh.edu.pl (D.G.); 2 Vesuvius Poland, Foseco Foundry Operation in Gliwice, ul. Leonarda da Vinci 5, 44-109 Gliwice, Poland

**Keywords:** grey cast iron, inoculation, primary austenite, degree of undercooling, tensile strength

## Abstract

Inoculation of cast iron has become a commonly used metallurgical process, which is carried out in a foundry in order to improve the mechanical properties of utility alloys. It consists in changing the physicochemical state of the melted alloy. This change is caused by the introduction of cast iron with a low ability to nucleate graphite, shortly before pouring a small mass of the substance—an inoculant that increases the number of active nuclei. It is also justified that the literature often connects an increase in the tensile strength UTS of the inoculated grey cast iron, with changes in the characteristics of the particles of graphite. However, in strongly hypoeutectic cast iron, in which a large number of primary austenite grains crystallize, the interdendritic distribution of graphite is usually the result. It also follows that the nature of the graphite precipitates is determined by the mutual relations between the interfacial distances in eutectic grains and the interdendritic distances in the grains of primary austenite occurring in the Fe–C alloys. The article presents the influence of the inoculant on the characteristics of the precipitation of primary austenite grains in relation to the sulphur content in grey cast iron with flake graphite. The study also showed that primary grains in grey cast iron have a great influence on mechanical properties, such as the tensile strength UTS. In this case, the key is to know the value of the degree of undercooling ΔT. The type of inoculant used affects the ΔT value. The study related the number of N primary austenite grains with the degree of undercooling ΔT and the tensile strength UTS with the number of primary austenite N grains.

## 1. Introduction

**Figure 1:**
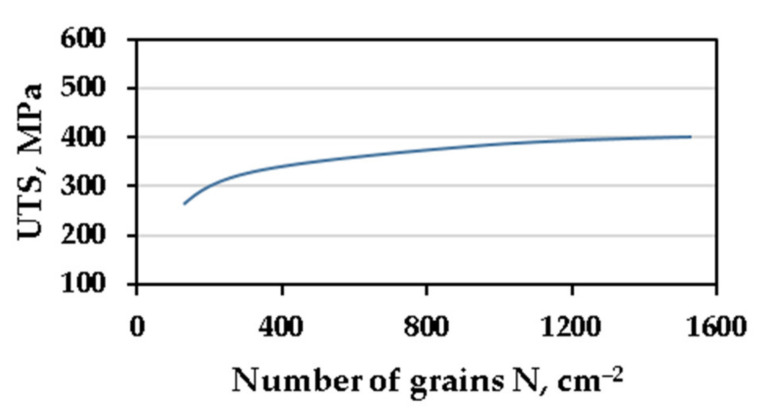
Dependence of grey cast iron tensile strength (UTS) on the number N of grains of graphite eutectic [8].

In industrial conditions, during the production of iron castings, the procedure of molten alloy inoculation (with low nucleation capacity) consists of introducing a small amount of the inoculant, which significantly increases the ability of the molten alloy to nucleate. As a result of increasing the number of substrates for heterogeneous nucleation of graphite, a much finer microstructure is obtained and, consequently, the properties of castings made of this iron are improved [1,2,3,4]. Undoubtedly, the most important indicator of the inoculation process assessment used in the cast iron technology is the increase in the number of eutectic grains [5,6,7,8]. Moreover, after the inoculation procedure, changes in the characteristics of the flake graphite particles are assessed. Graphite with an interdendritic distribution disappears in the structure of the inoculated cast iron, and an evenly distributed in flake graphite appears. In addition, the value of the degree of undercooling ΔT during the crystallization of the graphite eutectic, and the chilling tendency also decreases. It can be stated that the changes introduced by the inoculation in the cast iron microstructure improve its mechanical properties, as shown in Figure 1.

**Figure 2:**
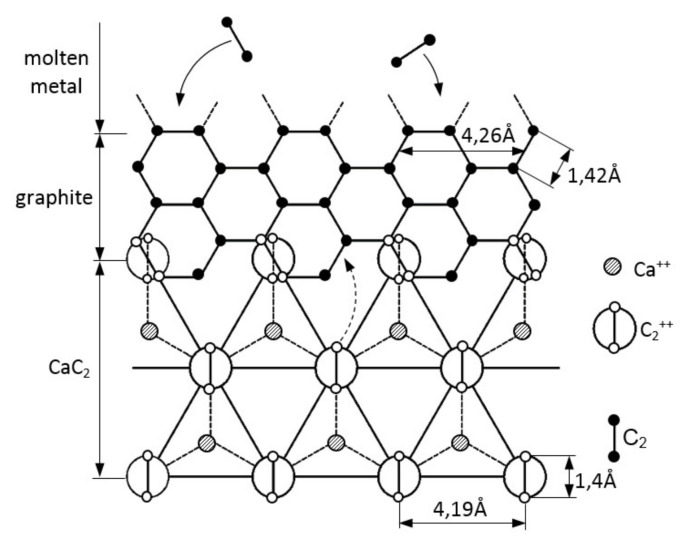
The mechanism of heterogeneous nucleation of graphite as a result of placing pairs of C–C atoms in the crystal lattice of CaC_2_ calcium carbide [4].

It should be noted that there is another important indicator of the course of inoculation, which, however, in practice is the most difficult to assess, namely: the characteristics of changes in the formation of primary austenitic dendrites. Primary austenite is the least investigated microstructural component of grey cast iron [9,10,11,12,13,14,15,16,17,18,19,20,21,22,23]. It is the first phase to be nucleated in the liquid metal and it grows in a dendritic manner, followed by growth of the eutectic phase. This is an important issue, as during the inoculation of grey cast iron, we influence not only the grains of the graphite eutectic, but also the number of the primary austenitic grains. In the literature there are at least four hypotheses concerning the problem of the cast iron inoculation process. However, it seems that the hypothesis that has the greatest justification in terms of industrial practice of inoculated iron by adding iron–silicon with small additions of elements of group II of the periodic table (Ca, Ba, Sr, etc.) and aluminum, is the hypothesis of B. Lux [3,4,5]. B. Lux, in his work, [3] proved that the introduction of the inoculant into the molten metal leads to crystallization in the liquid of carbides (MC_2_) with ionic bonds such as CaC_2_, BaC_2_, and SrC_2_. This also applies to other types of carbide: Al_4_C_3_ and Al_2_C_3_ [24,25]. These carbides act as substrate for heterogeneous nucleation of graphite. In practice, complex inoculants of the FeSi type are used in foundry, which includes about 75% silicon and small additions (up to a few percent by mass) of simple inoculants (for example: Ca, B, and Al). An exemplary mechanism of graphite nucleation on a CaC_2_ substrate is shown in Figure 2.

Simple inoculants are designed to affect graphite according to the mechanism shown in Figure 2, while the high content of silicon is responsible for the formation of zones locally saturated with this element in the molten alloy, which is the reason for the nucleation of the primary graphite and the intensification of the effect of calcium carbide CaC_2_.

It cannot be ruled out that standard inoculants introduce to liquid metal FeSi_2_ phase may be a suitable base for nucleation of primary austenitic dendrites [26]. In addition, in these types of complex inoculants which are used in the technology of obtaining inoculated cast iron, the following elements have been used: Bi, Al, La and other rare earth elements, and Ti, which undoubtedly form substrate for heterogeneous nucleation austenitic grains in the liquid alloy. Based on the industrial practice of cast iron foundry, it appears that the inoculation process considers the influence of the reagents on the grains of graphite eutectics, while the influence of this treatment on primary crystallization, i.e., on the grains of primary austenite, has been largely ignored to date. Understanding the principles of the crystallization of primary austenitic grains is crucial for the optimal treatment of cast iron inoculation. Moreover, in inoculated cast iron, the sulphur content is required at the level of 0.05–0.08% mass. With this sulfur content, the value of the tensile strength UTS is greater than 250 MPa. If the sulfur content in cast iron is below 0.05%, then the inoculation process does not proceed efficiently and the tensile strength UTS values are below expectations and the required 250 MPa. Undoubtedly, reduced sulfur content in the molten metal affects the number of primary austenite grains, a smaller number of which reduces the strength properties of cast iron. To eliminate this disadvantage, the liquid alloy should be enriched with sulphur, or the primary grains of austenite should increase their number. The first solution is difficult as it exists within a narrow range of the expected low sulfur content, i.e., 0.05–0.08% mass S. The second solution is related to the introduction of a special inoculant to affect the number of primary austenite grains and the comparison of the obtained strength properties of UTS in cast iron at different sulfur levels. The second is the purpose of this work and is not used in industrial conditions. In addition, the article develops an equation to predict the value of UTS depending on the degree of undercooling ΔT that can be measured during the casting production process.

## 2. Methodology

The tests were carried out in a medium-frequency induction furnace with a crucible capacity of 15 kg (Mammut type A-15). The metal charges consisted of Sorelmetal pig iron, steel scrap, technically pure silicon, ferromanganese, and iron sulphide. The inoculation of cast iron was carried out with the use of a Fe–Si inoculant (the chemical composition has been presented in Table 1) in the amount of 0.4% mass, iron powder—0.2% mass and fine particles steel scrap in the amount of 0.2% mass.

Chemical analysis of cast iron were performed using a HILGER spectrometer (Sterling, Margate, England). The average chemical composition of the tested cast iron was (% mass): 2.90–3.10% C, 1.85–2.05% Si, 0.45–0.55% Mn, 0.01–0.02% P, 0.01–0.03% Cr, 0.01–0.03% Ni, 0.01–0.03% Cu, and 0.01–0.03% Mo. Cast iron melts were carried out with a sulphur content at a level of 0.02% or 0.08%.

The melting procedure was as follows: after melting, the alloy was superheated to a temperature of 1490 °C and held at this temperature for about 120 s. The additional inoculants—iron powder or fine particles of steel scrap—were introduced into the bath at the temperature of 1460 °C. The standard inoculants A, B, and C were also introduced into the liquid alloy when the temperature dropped to the value of 1430 °C. Standard ⌀ 30 mm test rollers were cast, in order to make samples for tensile strength UTS tests and samples for metallographic specimens.

**Figure 3:**
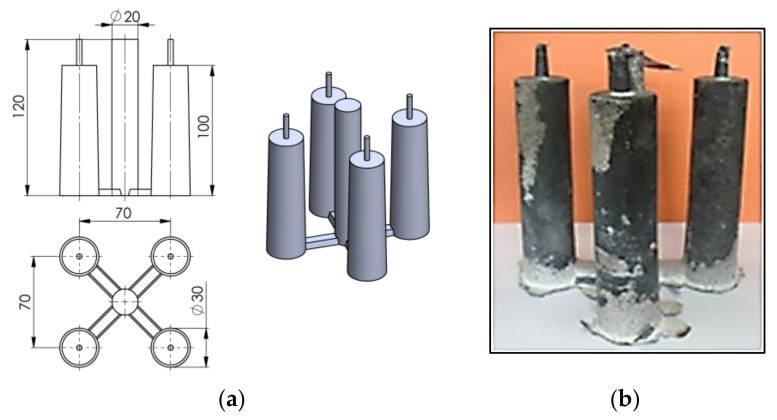
Test samples: model dimensions [27] (**a**) and appearance after heat treatment (**b**).

As a result of the research, it was found that the introduction of additional inoculants in the form of Fe powder or fine particles of steel scrap, shortly before pouring into the mold, increases the strength properties of cast iron with a low sulphur content. However, the metallographic tests carried out did not reveal the microstructure of primary austenitic grains (and the microstructure revealed by Nital etching is similar for samples with different sulfur contents). For this purpose, the DAAS method [10] was used. In order to determine the optimal heat treatment parameters, three initial heatings were carried out with different temperature values as shown in Table 2. It turned out that for the test samples shown in Figure 3 primary grains revealed in microstructure was obtained only for the procedure No. 3. and this very procedure was used in further research.

The first stage of heat treatment was carried out in a laboratory muffle furnace of the type: FCF 7SM, by the Czylok company (Jastrzębie-Zdrój, Poland), whereas austenitization was carried out in a laboratory salt bath. For further tests, a specially designed cast of four rollers with a diameter of 30 mm was used (Figure 3a), made with the technology of self-hardening loose sands with furfuryl resin.

Samples for strength and metallurgical tests were made from the bars. During the melting process, two sets of castings were cast (Figure 3b), one for grey cast iron tests, the other was subjected to DAAS heat treatment, and then the primary structure was tested. Controlled recording of temperature changes was carried out using a Pt-PtRh10 thermocouple, placed centrally along the axis of one of the cast bars.

### 2.1. Evaluation of the Grain Number of Primary Austenite

**Figure 4:**
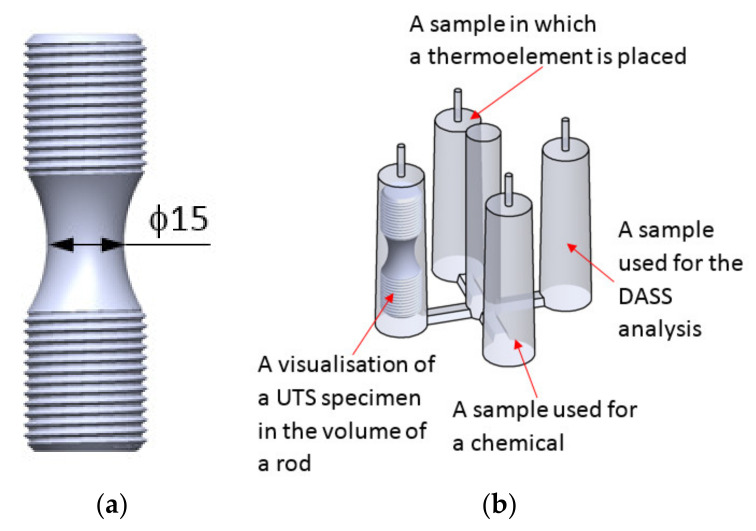
A scheme of the test sample: (**a**) a standard tensile specimen and (**b**) destination of all samples.

**Figure 5:**
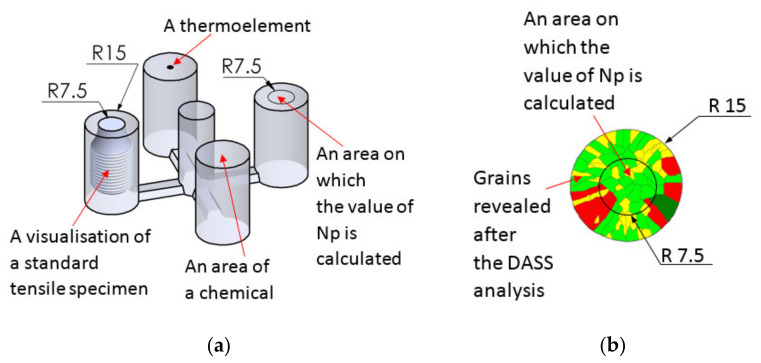
A cross-section of the sample casting (**a**) and an example of a cross-section of a rod with grains revealed by the DASS analysis (**b**).

The UTS tensile strength is performed on the specimen (Figure 4a). During the measurement, only the grains from the center of the rod (area with a diameter of 15 mm) take place in the examination. For a better illustration of the problem, the tensile specimen was made from a shaft (Figure 4b) which has been heat treated in DAAS method. The actual diameter of the surface of the specimen which takes part in the evaluation of the tensile strength is ⌀ 15 mm (Figure 5a). That is why the value of N_P_ is calculated only from the area marked by the circle in Figure 5a,b. Remaining material (outside the circle) was removed during the tensile sample making. In this article, we use two designations for the number of grains, i.e., N_A_ and N_P_. N_A_ is the number of primary austenite grains which was counted on the surface of a 30 mm diameter sample. While N_P_ is the number of primary austenite grains which was counted on the surface involved in the UTS tensile test—15 mm diameter in the center of the sample.

### 2.2. Procedure of Direct Austempering after Solidification

**Figure 6:**
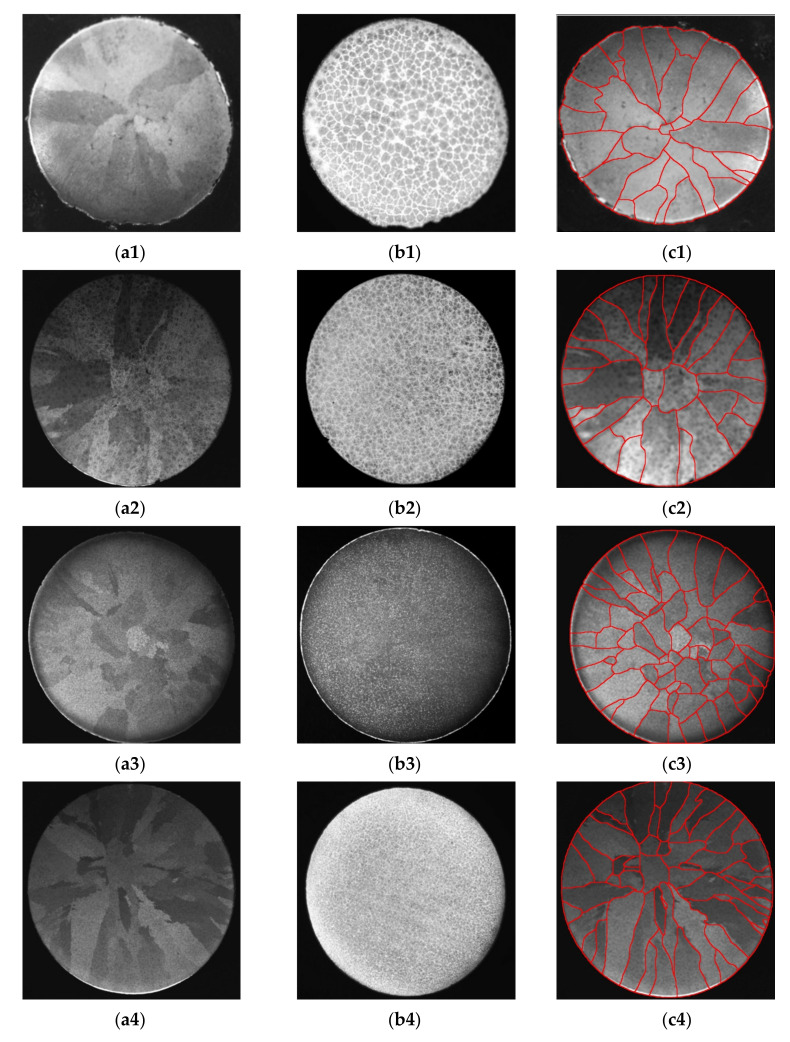

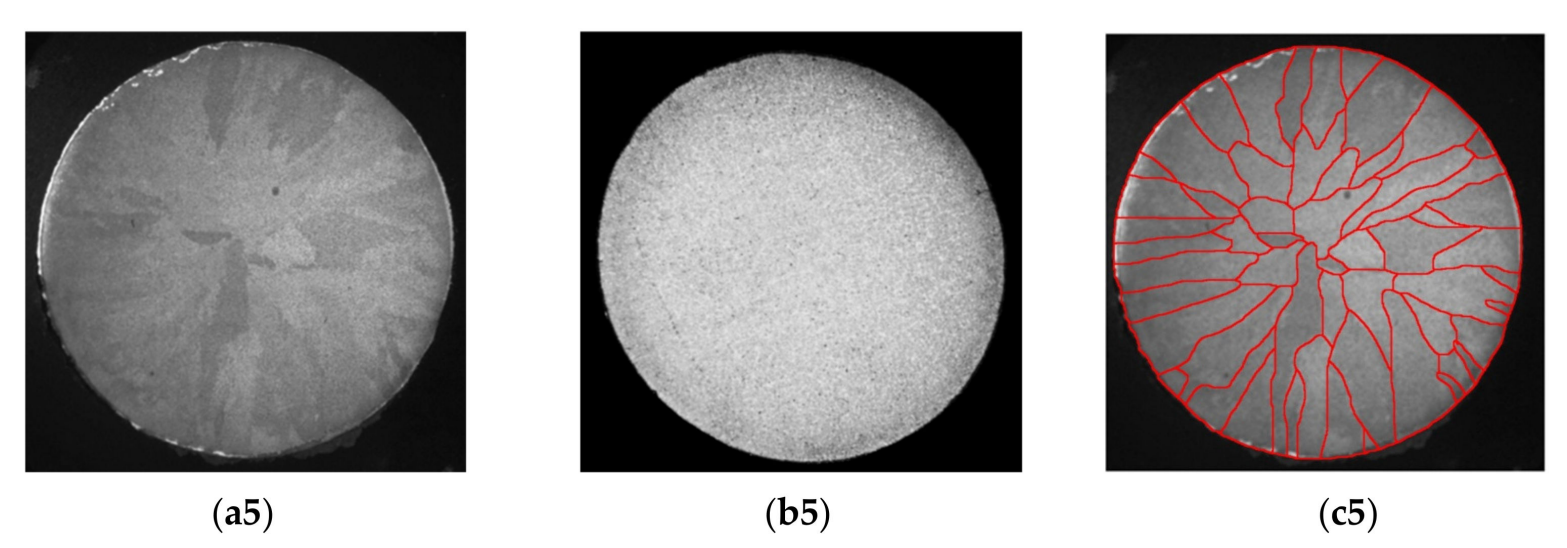
Appearance of primary austenite grains (**a**) and graphite eutectic grains (**b**) and example of use the so-called “mapping” of primary austenite grain boundaries in a grey cast iron sample after heat treatment with the DAAS technique (**c**); melts (Table 3): Z1—(**a1**–**c1**); Z2—(**a2**–**c2**); Z3—(**a3**–**c3**); Z4—(**a4**–**c4**), and Z5—(**a5**–**c5**); the actual diameter of the samples—30 mm.

In DAAS (Direct Austempering After Solidification) method, the austenite is retained in the structure and preserve the crystallographic orientation that was created during the crystallization of the casting. The procedure for this heat treatment is as follows: after pouring molten metal into a casting mold, the casting is knocked out of the mold when its temperature is about 950 °C, then the casting is transferred to an oven at 920 °C and held there for about 30 min. Next, the casting is isothermally hardened in a molten salt bath at 400 °C and kept there for 90 min. After keeping in the specified time the casting is cooled in air until it reaches room temperature. As a result of this heat treatment, the final microstructure of the sample consists of a mixture of ferrite and austenite. It should be added that under different angles of observation of a grey cast iron metallographic sample, a eutectic structure is observed, while under a different angle of observation, a primary austenite structure can be seen on the surface of the sample. An example of macrostructure obtained in the study is shown in Figure 6. The surfaces of the metallographic specimens were not etched. This is the state after heat treatment and polishing of the sample surface.

## 3. Test Results and Evaluation of the Primary Structure in Cast Iron Samples

The work carried out a series of melts, the chemical composition of which, and the metallurgical procedures used, are presented in Table 3. Inoculants containing barium (A), lanthanum (B), and titanium (C) were used. The chemical compositions of these inoculants are presented in Table 1. In this study, we wanted to reveal the structure of the primary austenite grains in samples Z1–Z5. This limitation is due to the difficult conditions of the experiment related to the disclosure of the primary austenite structure in DAAS method. The casting mold was poured with molten metal, knocked out as soon as possible and quickly introduced into molten salt bath. The remaining Z6–Z8 melts were intended to find an alternative inoculant for reduced sulphur cast iron. This was performed in the case of Z6B melt but with the condition of increasing the final silicon content in the molten metal. In Z6A–Z6B melts addition of iron–silicon (FeSi) were used without other inoculants. Z9–Z11 melts were used to compare the effect of inoculation on the UTS tensile strength value in increased sulfur content in cast iron.

As a result of the DAAS method, the exemplary microstructure shown in Figure 6 were obtained, showing the primary grains of austenitic dendrites, and eutectic grains were also revealed in the same metallographic specimens.

The study shows that the inoculation proposed in this paper for cast iron with reduced and increased sulfur content gives noticeable effects of increasing UTS tensile strength. In the case of comparing the same form of modification for cast iron with reduced (Z4) and increased (Z10) sulfur content, in the latter case the UTS was higher by 30 MPa. In the case of the Z11 melt (inoculant A), the value of the UTS tensile strength is 46 MPa higher compared to the cast iron from the Z3 melt (inoculant A). However, cast iron from the Z3 melt does not reach the normative value specified in the standards, i.e., 250 MPa. The conducted research has shown that when using standard inoculants for cast iron with reduced sulfur content, obtaining UTS at the level of 250 MPa is difficult (inoculant C works; A and B were unsuccessful), while obtaining UTS at the level of 300 MPa is impossible in all described cases. The introduction of iron powder as an additive to the standard inoculant (for cast iron with reduced and increased sulfur content) allowed us to obtain UTS values of over 300 MPa. The melting of Z6A and Z6B was carried out to test ferrosilicon (without any additives) as an inoculant for cast iron with reduced sulphur content. It was found that during the Z6B melting, satisfactory UTS values of the order of 350 MPa were obtained. In Z5 melt (C-type inoculant applied), 284 MPa UTS was obtained. The inoculant in this case included titanium (9–11 % mass), which undoubtedly affected the number of primary austenite grains. However, the replacement of Fe powder with fine steelscrap does not lead to achieving the required minimum UTS value (Z7 melt). A similar phenomenon occurred in the case of application of inoculant type B (Table 1) with lanthanum addition (Z8 melt).

## 4. Thermal Analysis of Tested Samples

**Figure 7:**
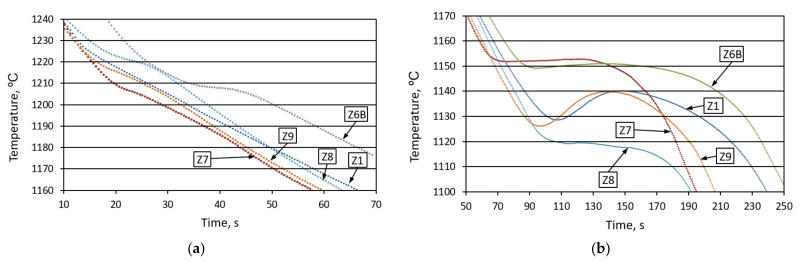
Examples of temperature changes in the center of a ⌀ 30 mm cast iron roller in the liquidus temperature range (**a**) and in the eutectic crystallization range (**b**).

Figure 7 shows an example of the measurements of the temperature value change in a cast iron roller with a diameter of 30 mm. Additionally, the graphs show the results of T liquidus and Te_min_ measurements, determined by thermal analysis.

**Figure 8:**
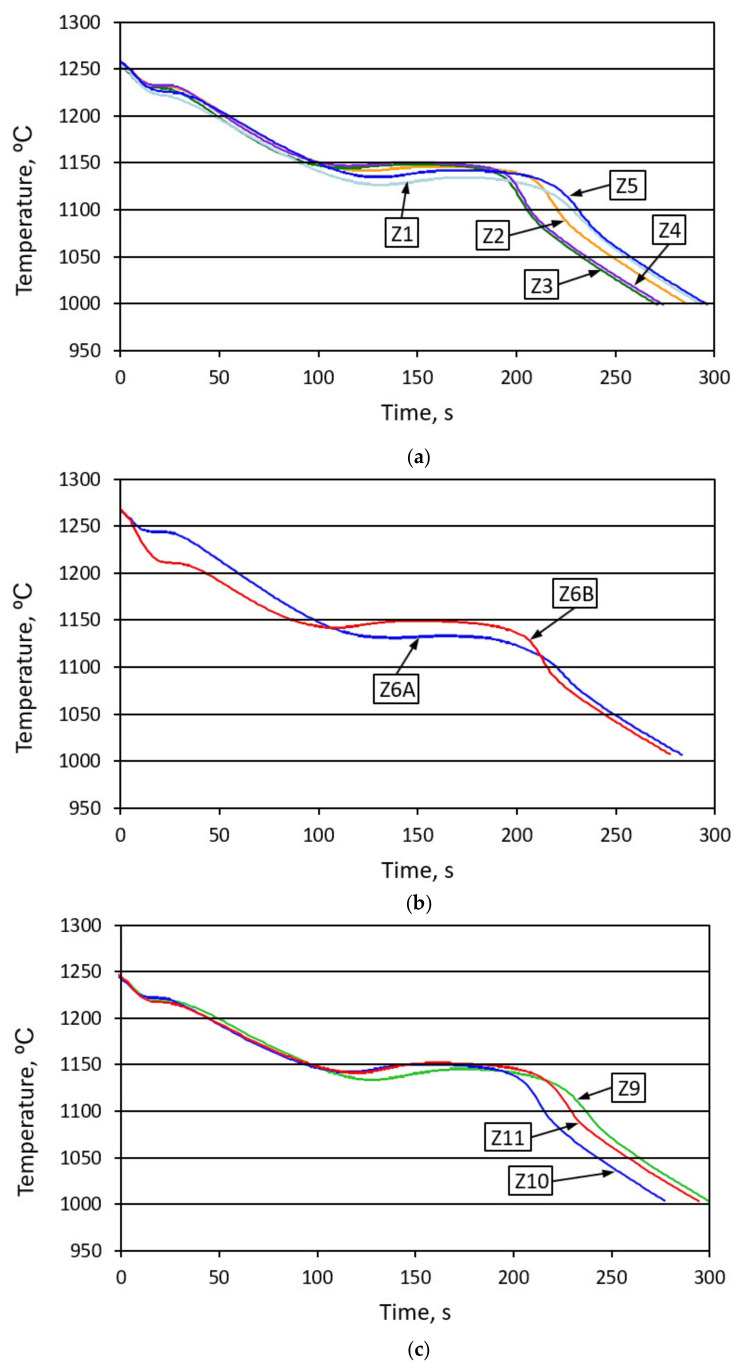
Summary of the cast iron crystallization and cooling curves for samples: Z1, Z2, Z3, Z4, and Z5—(**a**), Z6A and Z6B—(**b**), and Z9, Z10, and Z11—(**c**) recorded by the thermal analysis system (color marking in accordance with Table 4).

Figure 8 shows the changes in the physicochemical state before and after inoculation with ferrosilicon (1.4% by weight). There, we can observe a slightly bigger influence of the ferrosilicon introduced on the liquidus temperature value, as well as the degree of undercooling ΔT for the crystallization of the primary austenitic grains and the graphite eutectic grains, compared to the heatings from Z1 to Z5. Table 4 presents the physicochemical parameters of the alloy.

In fact, these are two different levels of physicochemical state, therefore the simultaneous analysis of melts Z6A and Z6B cannot be carried out in any way with melts Z1 to Z5. The reason is introduce large amounts of silicon, which changes the physicochemical state of the molten metal, in two ways. During the Z1–Z5 melts, silicon was mainly introduced into the metal charge. During the Z6A and Z6B melts, silicon was introduced to molten metal as the inoculant. It should be remembered that the physicochemical state of the liquid alloy is determined by its physical properties (viscosity, surface, or interfacial tension), the presence of ordered complexes of atoms (clusters) and non-metallic inclusions.

## 5. Modeling of Thermodynamic Parameters of the Tested Cast Iron Using the Themo-CALC Program

The calculations of the temperature of phase transformations for the cast iron with a given chemical composition presented in Table 5, were made using the Thermo-CALC program (ver. 2019b), based on the CALPHAD method.

The knowledge of the phase equilibrium diagrams of alloys allows us to understand the processes mechanisms in the formation of the microstructure of alloys during crystallization and heat or thermochemical treatment. This method is used to model thermodynamic phase parameters and simulate the behavior of complex, multicomponent, multi-phase systems. The basis of the modeling is the calculation of the “Gibbs energy” for the individual phases, depending on temperature and chemical composition.

Equilibrium temperature in Table 5 determines at which temperature there is a change in phase composition. Phases before the “=” sign are stable for higher whereas after the “=” sign are stable for lower temperatures

**Figure 9:**
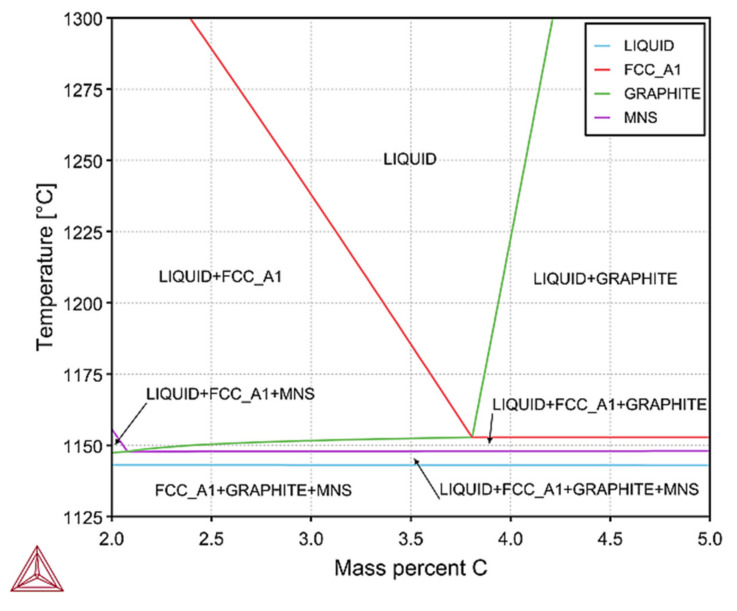
The part of the equilibrium phase diagram calculated by the Thermo-CALC program; FCC_A1—Face Centered Cubic phase (austenite), and MNS—MnS.

**Figure 10:**
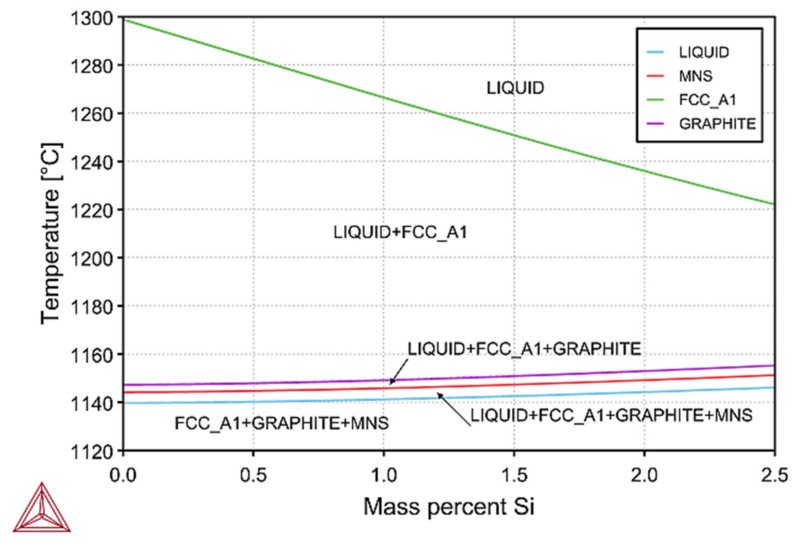
The silicon addition effect from melts No. Z1–Z8 calculated by the Thermo-CALC program; FCC_A1—Face Centered Cubic phase (austenite), and MNS—MnS.

These dependencies make up thermodynamic databases, the accuracy of which depends on the reliability of Thermo-CALC calculations. Thermodynamic calculations by the CALPHAD method, are a convenient way to obtain phase diagrams (polythermal sections), as seen in Figure 9 and Figure 10. Results were obtained using Thermo-CALC Software ver. 2019b with thermodynamic database: TCFE7 and with assumption of equilibrium calculations. For the chemical composition of cast iron from melts Z1 to Z8, the calculated fragment of the phase equilibrium system of Fe–C alloys is shown in Figure 9.

This figure shows a polythermic cross-section for the five-component Fe–C–Si–Mn–S system. This system shows that the components of the phase of the cast iron structure nucleate, and growth was in the following order: austenite, graphite, and the last, manganese sulphide—MnS. Therefore, in this type of cast iron, the possibility of nucleation of graphite grains on MnS is excluded. On the other hand, the change in the silicon content does not affect this process, and with a low value of the sulphur content in cast iron, graphite does not nucleate on the MnS particles, which was confirmed in Figure 10.

## 6. List of Crystallization Parameters

For the purpose of determining the maximum degree of undercooling ΔT* for primary austenite, the formula determined by [3,28] was used:

(1) ΔT* = T_γ_ − T_γ_*

T_γ_ = 1636 − 113 × (C + 0.25 × Si + 0.25 × P), T_γ_*—measured with a thermocouple (T_liquidus_ in Table 4); C, Si, P—% mass.

An analysis of the degree of undercooling was carried out on the basis of simulation in the Term-CALC—ΔT** program. A similar comparison was made to determine the maximum degree of undercooling ΔTe for graphite eutectics [28]:

(2) ΔTe = T_e_ − T_e_*

T_e_ = 1153.97 + 5.25 × Si − 14.88 × P, T_e_*—measured with a thermocouple; Si, P—% mass.

The Poisson–Voronoi formula was used to determine the number of grains per cm^3^:

(3) Nv = 0.5680 × N_A_^3/2^, 1/cm^3^

where N_A_ is the number of grains, 1/cm^2^.

The stereological Equation (3) can be used to calculate the spatial grain number Nv, which should give the average number of grains of primary austenite per unit volume. During the calculations, the following assumptions were made that the spatial grain configurations follow the so-called Poisson–Voronoi model [29]. This formula is used to calculate the number of graphite eutectic cells [30]. However, it can be useful for calculating the number of primary austenite grains, especially for the middle area of the sample, where we have equiaxial grains (N_P_). Summary crystallization parameters of alloys are presented in Table 6.

Table 6 summarizes the values of the crystallization parameters of the alloys for further analysis and shows one basic conclusion: that the value of the degree of undercooling ΔT for the crystallization of primary and eutectic grains may differ significantly (even by about 10 K) depending on the adopted calculation method. Therefore, for further research, it was decided to use formula No. 1 to calculate the degree of undercooling ΔT for the crystallization of primary austenitic grains.

## 7. Relation of the Degree of Undercooling ΔT and Tensile Strength UTS with the Number of Grains of the Primary Austenite N_A_

**Figure 11:**
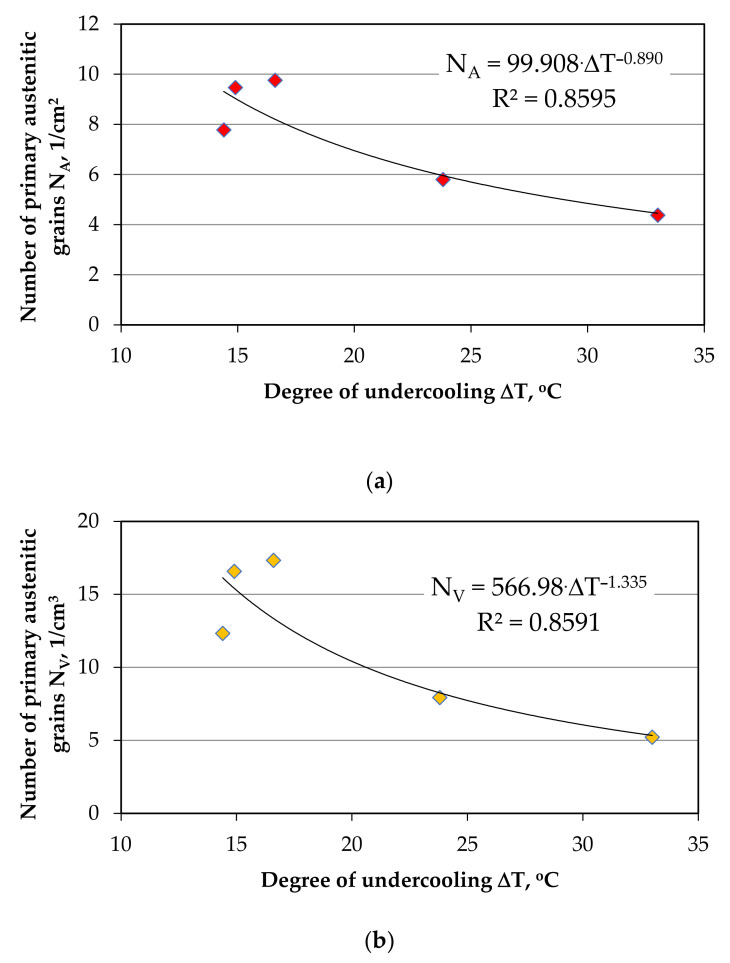
The influence of inoculation on the degree of undercooling ΔT and the number of primary austenitic grains N_A_/cm^2^ (**a**) and N_V_/cm^3^ (**b**) for a similar eutectic saturation coefficient S_c_ in Z1–Z5 melts.

As a result of the research, it can be concluded that grey iron castings with low sulphur content have a reduced tensile strength UTS and have defects, such as microporosity. Figure 11 present the effect of the degree of undercooling ΔT on the number of primary austenite grains N_A_. This summary requires a comment as it should be noted that the research considered, concerns the problem of inoculation of the molten alloy, characterized by a similar eutectic saturation coefficient—S_c_. Hence, the correlation shown in Figure 11 is possible.

As can be expected, each melt is characterized by a separate physicochemical state; therefore, one must take into account some non-calculable error related to the above tests. However, the research shown in Figure 11, clearly shows that introducing substrate for heterogeneous nucleation of primary austenite grains, increases their number and thus reduces the degree of undercooling ΔT, according to the following equations (for a similar eutectic saturation coefficient Sc):

(4) N_A_ = 99.908 × ΔT^−0.890^, 1/cm^2^

(5) N_V_ = 566.98 × ΔT^−1.335^, 1/cm^3^

**Figure 12:**
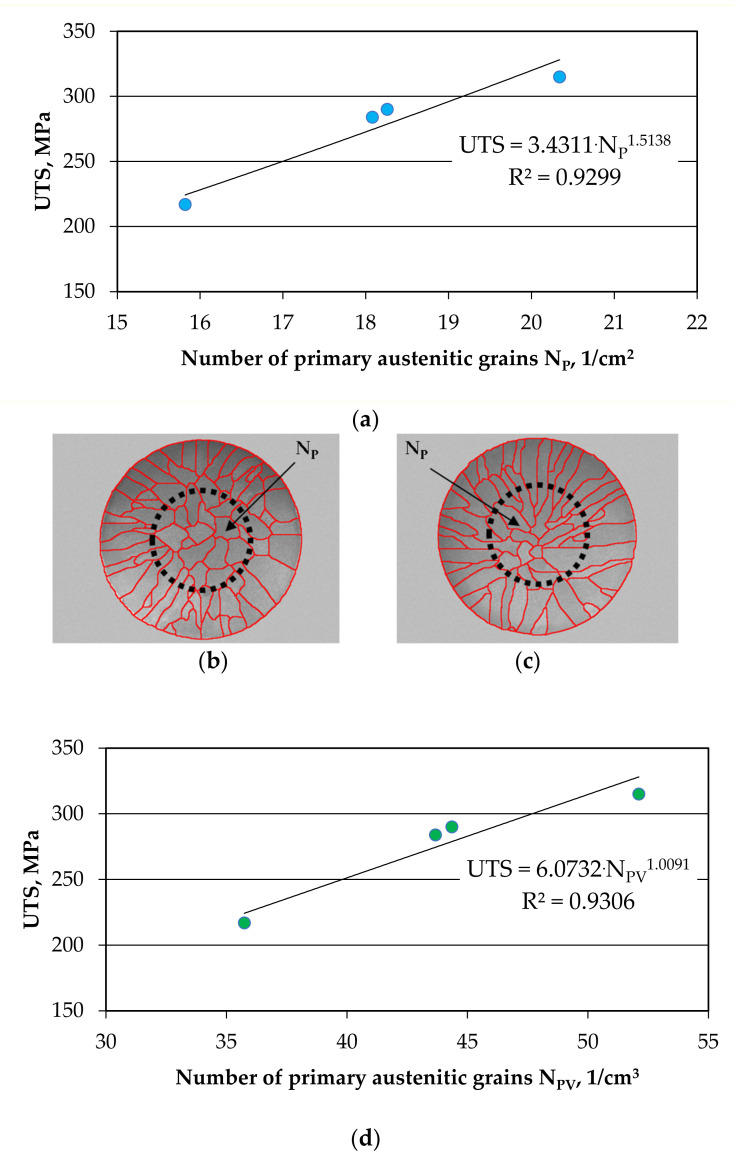
The influence of inoculation on the number of primary austenite grains N_P_/cm^2^ (**a**) and N_PV_/cm^3^ (**d**) on the value of tensile strength UTS for a similar eutectic saturation coefficient Sc (Z2–Z5 melts); the number of primary grains N_P_ for sample Z3 (**b**) and Z5 (**c**) from the UTS strength test measuring area ⌀15 mm.

If we take into account the UTS value of the grey iron samples given in Table 3 and link them with the number of primary austenitic grains N_A_, it is revealed that it is not possible to obtain any correlation. Figure 12 shows the courses of Equations (6) and (7) against the background of the measuring points.

If we assume the diameter of the strength sample (in the case of tests, the actual diameter of the sample rupture was up to 15 mm (see Figure 12b,c versus Figure 4 and Figure 5) and calculate the number of grains N_P_ for cast iron samples on such a diameter surface, then we will obtain the following equations:

(6) UTS = 3.4311 × N_P_^1.5138^, MPa

(7) UTS = 6.0732 × N_PV_^1.0091^, MPa

## 8. Conclusions

The mechanical and functional properties of cast iron castings are directly influenced by its microstructure, which consists of graphite and a metal matrix. After the study, it must be concluded that the sizes and numbers of austenitic primary grains are crucial for obtaining good mechanical properties of defect-free iron castings. This is especially true for inoculated low-sulphur grey cast iron, in which the comminution of the austenitic primary grains increases its tensile strength (UTS). The study shows that the inoculation proposed in this paper for cast iron with reduced and increased sulfur content improves UTS tensile strength. In both cases, the addition of iron powder is required to obtain a UTS value of 300 MPa or more. This demonstrates that additional nuclei of primary austenite crystallization must be initiated. In addition, the study shows that the use of iron–silicon (without other inoculants) as an inoculant of cast iron with reduced sulfur content leads to a high UTS value of 350 MPa, while keeping the condition of increased silicon content in cast iron to 2.10 wt% (melt Z6B versus melt Z6A in Table 3).

The research on the structure formation in inoculated grey cast iron with low sulphur content allowed to calculate of Equations (4) and (5) linking the number of primary austenitic grains N_A_ with the degree of undercooling ΔT. These studies also allowed to link the number of primary grains NP with the tensile strength UTS of cast iron, which was presented in Equations (6) and (7). It should be noted that the above-mentioned equations predict the value of the considered parameters for the eutectic saturation coefficient (Sc) of 0.78 for cast iron rollers with a diameter of ⌀ 30 mm, poured into self-hardening sand molds.

As a result of the above analysis of conducted research, the following conclusions can be presented:

- In the inoculation of grey cast iron with low sulphur content, the iron particles (substrate for heterogeneous nucleation) affect the process of crystallization of the primary austenitic grains.
- Iron particles can be introduced into the molten alloy in the form of iron powder, as well as ferroalloy FeSi.
- Knowing the degree of undercooling of primary austenite grains, the number of primary austenite grains N_A_ can be calculated using the following equation:

N_A_ = 99.908 × ΔT^−0.890^, 1/cm^2^

- Knowing the number of primary austenite grains N_P_ (in area on the actual diameter of the sample rupture in UTS method), the UTS can be calculated using the following equation:

UTS = 3.4311 × N_P_^1.5138^, MPa

## Figures and Tables

**Table 1:** Chemical composition of complex inoculants.

Inoculant	Si	Ca	Al	La	Ba	Ti	Fe
	% mass	% mass	% mass	% mass	% mass	% mass	% mass
A	64–70	1.0–2.0	0.8–1.5	-	2.0–3.0	-	remain
B	40–45	-	1.0–1.5	1.5–1.8	-	-	remain
C	50–55	max. 1	max. 1	-	-	9–11	remain

**Table 2:** Temperatures used during heat treatment with the DAAS method.

No.	Oven Temperature, °C	Salt Bath Temperature, °C	Microstructure
1.	880	360	Pearlitic matrix
2.	900	360	Ausferrite coating
3.	920	400	Primary grains revealed

**Table 3:** Conducted melts, their chemical composition and tensile strength UTS value.

No.	Cast Iron	Chemical Composition, % mass						UTS, MPa
		C	Si	Mn	P	S	S_c_ *	Average of Three Measurements
Z1	Reference cast iron	2.92	1.65	0.38	0.03	0.014	0.78	Whitened sample
Inoculation treatment								
Z2	0.4% Fe powder	2.91	1.66	0.37	0.04	0.013	0.78	290
Z3	0.4% Inoculant A	2.92	1.91	0.36	0.04	0.013	0.79	217
Z4	0.2% Fe powder and 0.4% Inoculant A	2.94	1.80	0.39	0.05	0.012	0.79	315
Z5	0.4% Inoculant C	2.96	2.00	0.41	0.03	0.011	0.81	284
Z6A	1.4% FeSi	2.94	0.85	0.31	0.03	0.013	0.74	210
Z6B		2.95	2.10	0.35	0.03	0.014	0.82	350
Z7	0.2% fine particles of steel scrap and 0.4% Inoculant A	2.96	2.05	0.41	0.037	0.013	0.81	197
Z8	Inoculant B	2.94	1.95	0.44	0.05	0.011	0.80	Whitened sample
Cast iron with increased sulfur content								
Z9	Reference cast iron with	3.02	1.61	0.40	0.04	0.09	0.80	Whitened sample
Inoculation treatment								
Z10	0.2% Fe powder and 0.4% Inoculant A	2.97	1.85	0.41	0.045	0.09	0.80	345
Z11	0.4% Inoculant A	2.97	1.87	0.40	0.04	0.09	0.80	263

* Eutectic saturation coefficient—S_c_ = C/(4.26 – 0.3 × Si – 36 × P); C, Si, P—% mass.

**Table 4:** The crystallization parameters according to thermal analysis.

No.	T_liquidus_	*Te
	°C	°C
Z1	1224.7	1131.3
Z2	1234.2	1145.9
Z3	1233.2	1148.7
Z4	1235.7	1151.4
Z5	1228.4	1139.2
Z6A	1243.5	1130.6
Z6B	1210.2	1141.6
Z7	1206.9	1152.1
Z8	1222.5	1122.0
Z9	1216.6	1133.1
Z10	1219.0	1141.3
Z11	1214.3	1140.5

*Te—crystallization temperature of the eutectic.

**Table 5:** The calculated values of the equilibrium crystallization temperature of the primary austenitic grains and the grains of eutectic graphite.

No.	Chemical Composition, % mass					T Liquid °C	T Equilibrium °C		
	C	Si	Mn	P	S				
							LIQ + FCC = LIQ + FCC + GRA	LIQ + FCC + GRA = LIQ + FCC + GRA + MNS	LIQ + FCC + GRA + MNS = FCC + GRA + MNS
Z1	2.92	1.65	0.38	0.030	0.014	1246.24	1151.76	1148.33	1143.51
Z2	2.91	1.66	0.37	0.040	0.013	1246.56	1151.54	1147.34	1141.07
Z3	2.92	1.91	0.36	0.040	0.013	1238.19	1152.66	1148.20	1141.87
Z4	2.94	1.80	0.39	0.050	0.012	1238.76	1151.80	1146.86	1138.87
Z5	2.96	2.00	0.41	0.030	0.011	1231.84	1153.26	1149.26	1144.58
Z6A	2.94	0.85	0.31	0.030	0.013	1269.52	1149.66	1145.76	1141.71
Z6B	2.95	2.1	0.35	0.030	0.014	1230.13	1153.86	1149.90	1145.07
Z7	2.96	2.05	0.41	0.037	0.013	1229.98	1153.21	1149.17	1142.98
Z8	2.94	1.95	0.44	0.050	0.011	1234.24	1152.25	1147.37	1139.16
X	X	X	X	X	X	X	LIQ + FCC = LIQ + FCC + MNS	LIQ + FCC + MNS = LIQ + FCC + GRA + MNS	LIQ + FCC + GRA + MNS = FCC + GRA + MNS
Z9	3.02	1.61	0.40	0.040	0.09	1233.37	1171.38	1149.84	1141.04
Z10	2.97	1.85	0.41	0.045	0.09	1231.17	1173.33	1150.62	1140.46
Z11	2.97	1.87	0.40	0.040	0.09	1230.88	1171.98	1150.84	1141.79

LIQ—liquid phase, FCC—Face Centered Cubic (austenite), GRA—graphite, and MNS—MnS.

**Table 6:** Summary crystallization parameters of alloys.

No.	CAST Iron	Austenite Crystallization				Eutectic Crystallization		
		N_A_/cm^2^ *N_P_*/cm^2^	N_V_/cm^3^ *N_PV_*/cm^3^	ΔT*, °C ΔT**, *°C*	T_L_, °C	Ne/cm^2^	ΔTe, K	Te
Z1	Reference cast iron	4.38 *6.78*	5.21 *10.02*	33.0 *25.0*	1224.7	57	30.9	1131.3
Inoculation treatment								
Z2	0.4% Fe powder	5.80 *18.26*	7.93 *44.35*	23.8 *15.0*	1234.2	104	16.2	1145.9
Z3	0.4% Inoculant A	9.76 *15.82*	17.32 *35.74*	16.6 *8.0*	1233.2	223	14.7	1148.7
Z4	0.2% Fe powder and 0.4% Inoculant A	7.78 *20.34*	12.32 *52.11*	14.4 *7.0*	1235.7	246	11.3	1151.4
Z5	0.4% Inoculant C	9.47 *18.08*	16.57 *43.67*	14.9 *6.0*	1228.4	156	24.8	1139.2
Z6A	1.4% FeSi	X	X	34.6 *26.0*	1243.5	32	27.1	1130.6
Z6B		X	X	31.4 *19.9*	1210.2	256	22.8	1141.1
Z7	0.2% steel sheet scrap and 0.4% Inoculant A	X	X	34.6	1206.9	-	12.1	1152.1
Z8	Inoculant B	X	X	23.4	1222.5	-	41.5	1122.0
Cast iron with increased sulfur content								
Z9	Reference cast iron with increased sulfur content	X	X	30.4	1216.6	63	28.7	1133.1
Inoculation treatment								
Z10	0.2% Fe powder and 0.4% Inoculant A	X	X	26.6	1219.0	283	21.7	1141.3
Z11	0.4% Inoculant A	X	X	31.0	1214.3	269	22.7	1140.5

N_A_—number of primary austenite grains on the total sample surface ⌀30 mm. N_P_—number of primary austenite grains on the central surface of the sample ⌀15 mm, i.e., the actual burst area of the specimen in the UTS tensile test, the UTS specimens are lathed out from 30 mm diameter roller—see Figure 10. N_PV_—volumetric number of primary austenite grains on the central surface of the sample ⌀15 mm, the result of applying the Poisson–Voronoi formula for N_P_. Te—crystallization temperature of the eutectic (measured). Ne—number of eutectic grains. ΔT*—the degree of undercooling was calculated using formula (1). ΔT**—the degree of undercooling was calculated using Thermo-CALC.

## Data Availability

Not applicable.
